# Antibodies against conserved amidated neuropeptide epitopes enrich the comparative neurobiology toolbox

**DOI:** 10.1186/2041-9139-3-23

**Published:** 2012-10-01

**Authors:** Markus Conzelmann, Gáspár Jékely

**Affiliations:** 1 Max Planck Institute for Developmental Biology, Spemannstrasse 35, Tübingen 72076, Germany

## Abstract

**Background:**

Neuronal antibodies that show immunoreactivity across a broad range of species are important tools for comparative neuroanatomy. Nonetheless, the current antibody repertoire for non-model invertebrates is limited. Currently, only antibodies against the neuropeptide RFamide and the monoamine transmitter serotonin are extensively used. These antibodies label respective neuron-populations and their axons and dendrites in a large number of species across various animal phyla.

**Results:**

Several other neuropeptides also have a broad phyletic distribution among invertebrates, including DLamides, FVamides, FLamides, GWamides and RYamides. These neuropeptides show strong conservation of the two carboxy-terminal amino acids and are α-amidated at their C-termini. We generated and affinity-purified specific polyclonal antibodies against each of these conserved amidated dipeptide motifs. We thoroughly tested antibody reactivity and specificity both by peptide pre-incubation experiments and by showing a close correlation between the immunostaining signals and mRNA expression patterns of the respective precursor genes in the annelid *Platynereis.* We also demonstrated the usefulness of these antibodies by performing immunostainings on a broad range of invertebrate species, including cnidarians, annelids, molluscs, a bryozoan, and a crustacean*.* In all species, the antibodies label distinct neuronal populations and their axonal projections. In the ciliated larvae of cnidarians, annelids, molluscs and bryozoans, a subset of antibodies reveal peptidergic innervation of locomotor cilia.

**Conclusions:**

We developed five specific cross-species-reactive antibodies recognizing conserved two-amino-acid amidated neuropeptide epitopes. These antibodies allow specific labelling of peptidergic neurons and their projections in a broad range of invertebrates. Our comparative survey across several marine phyla demonstrates a broad occurrence of peptidergic innervation of larval ciliary bands, suggesting a general role of these neuropeptides in the regulation of ciliary swimming.

## Background

Antibodies that show specific immunoreactivity across a broad range of species are valuable tools for comparative neuroanatomy in non-model organisms. For example, antibodies against serotonin commonly label cell bodies and their projections, allowing comparative studies of neurodevelopment and neuroanatomy across diverse species and phyla [1]. Another commonly used antibody is that against FMRFamide, a neuropeptide first discovered in molluscs [2,3]. Similar RFamide neuropeptides were later found to be widespread among eumetazoans [4-6]. A pioneering work reported the development of antibodies against the conserved amidated dipeptide motif RFamide [7]. This RFamide and other FMRFamide antibodies have been extensively used in invertebrate neuroanatomy, owing to the broad distribution of RFamide-like peptides [8]. The RFamide antibody labels distinct neuronal subsets and their projections, and can be applied as a neuronal marker to increase morphological resolution in complex adult tissues [9], or to reveal aspects of nervous system development and organization, allowing the clarification of phylogenetic relationships within phyla [10-12] or the study of nervous system evolution between related groups [13].

Neuropeptides are signalling molecules that are translated as precursor molecules, typically consisting of an N-terminal signal peptide and multiple copies of similar peptide motifs, flanked by dibasic cleavage sites (Lys and Arg residues). The precursor is cleaved and often further modified to yield shorter active neuropeptides [14,15]. α-amidation is the most common post-translational modification, where a C-terminal glycine is enzymatically converted into an amide group. This modification protects the small peptides from degradation and is critical for receptor binding [16-18]. Amidation is also thought to confer high immunogenic potential to short neuropeptides [19-21] and antibodies raised against amidated peptides are highly specific for the amidated peptide moiety [21]. Changes in hydrogen bonding capability caused by the amide group may lead to the improved receptor binding and increased immunogenicity of C-terminally amidated peptides [22].

The C-terminal residues in amidated neuropeptides are often highly conserved across different species and even phyla [23]. We reasoned that, like the RFamide antibodies, other dipeptide antibodies could also potentially be used as neuronal markers across a wide range of species. Here we report the development of specific neuronal antibodies against the amidated dipeptide motifs of five conserved neuropeptides, DLamide, FVamide, FLamide, GWamide and RYamide. We show that these antibodies recognize specific subsets of neurons and their projections in cnidarian, annelid, mollusc, bryozoan and crustacean larvae. Furthermore, our antibody stainings reveal that the neuropeptidergic innervation of locomotor cilia is a general feature of ciliated larvae.

## Methods

### Generation of polyclonal neuropeptide antibodies

The amidated peptides, coupled to an adjuvant (lipoadjuvant Pam3) via an N-terminal cysteine (CRYamide, CGWamide, CFVamide, CFLamide, CDLamide), were used to immunize rabbits. Sera were affinity-purified on the respective peptide epitopes using a SulfoLink resin (Thermo Scientific, Rockford, USA) that allows the coupling of cysteine containing peptides via a disulphide bond. After coupling of 1 mg peptide epitope to 2 ml resin in Coupling Buffer (CB; 50 mM TRIS pH 8.5, 5 mM EDTA), the resin was washed three times with 10 ml CB. Excess reactive sites were blocked by incubating the resin in 2 ml 50 mM cysteine for 45 min, followed by three washes with 1 M NaCl and three washes with 25 ml phosphate buffered saline (PBS). Next, 25 ml serum was applied to the resin and this was incubated overnight to allow antibody binding. After flow-through of the serum, the resin was washed five times with 25 ml PBS followed by a wash with 15 ml 0.5 M NaCl/PBS and again twice with 10 ml PBS. The antibodies were eluted and fractionated with eight times 1 ml of 100 mM glycine pH 2.7, eight times 1 ml of 100 mM glycine pH 2.3 and eight times 1 ml of 100 mM glycine pH 2.0. The fractions were neutralized by directly collecting them in an adequate volume (about 40, 75 and 95 μl for the different pH solutions) of 1 M TRIS–HCl pH 9.5. The protein concentration of each fraction was determined, and the first two fractions of the pH 2.7 peak (usually fractions 2 and 3) were discarded, since these contained the lowest affinity antibodies. The peak fractions and the end-of-peak fractions were pooled, and concentrated, if necessary, using Vivaspin centrifugation tubes with a molecular weight cut-off of 10 kDa (Sartorius, Göttingen, Germany). Antibodies were stored in 50% glycerol at −20°C for mid-term (up to 1 year), and −80°C for long-term storage. A detailed protocol is available [24].

### Immunohistochemistry

For immunostainings, larvae were fixed in 4% formaldehyde in PTW (PBS + 0.1% Tween-20) for 2 h and stored in 100% methanol at −20°C until use. After stepwise rehydration to PTW, samples were permeabilized with proteinase-K treatment (100 μg/ml in PTW, for 1 to 3 min). To stop proteinase-K activity, larvae were rinsed with glycine buffer (5 μg/ml in PTW) and post-fixed in 4% formaldehyde in PTW for 20 min followed by two 5 minwashes in PTW and two 5 minwashes in THT (0.1 M TRIS–HCl pH 8.5 + 0.1% Tween-20). Larvae and antibodies were blocked in 5% sheep serum in THT for 1 h. Primary antibodies were used at a final concentration of 1 μg/ml for rabbit neuropeptide antibodies and 0.5 μg/ml for mouse anti-acetylated tubulin antibody (Sigma, Saint Louis, USA) and incubated overnight at 6°C. Weakly bound primary antibodies were removed by two 10 min washes in 1 M NaCl in THT, followed by five 30 min washes in THT. Larvae were incubated overnight at 6°C in the dark in 1 μg/ml anti-rabbit Alexa Fluor® 647 antibody (Invitrogen, Carlsbad, CA, USA) and in 0.5 μg/ml anti-mouse FITC antibody (Jackson Immuno Research, West Grove, PA, USA) and then washed six times for 30 min with THT-buffer, and mounted in 87% glycerol including 2.5 mg/ml of the anti-photobleaching reagent 1,4-diazabicyclo[2.2.2]octane (Sigma, St. Louis, MO, USA). *Pecten* larvae were additionally treated with 4% paraformaldehyde in PBS with 50 μM EDTA pH 8.0 for 1 h to decalcify their shells before the immunostaining procedure (performed as described previously). For cnidarian larvae, we also used a mouse anti-tyrosylated tubulin antibody (Sigma, Saint Louis, USA) at 1 μg/ml. For immunostaining with multiple rabbit primary antibodies in the same sample, antibodies were directly labelled with a fluorophore using the Zenon® Tricolour Rabbit IgG Labelling Kit (Invitrogen, Carlsbad, CA, USA) and used in combination with mouse anti-acetylated tubulin antibody.

For blocking experiments, we pre-incubated the antibodies in 5 mM of the respective full-length *Platynereis* peptides (YYGFNNDLamide, AHRFVamide, AKYFLamide, VFRYamide, RGWamide) for 2 h before immunostainings.

### Microscopy and image processing

Images were taken on an Olympus Fluoview-1000 confocal microscope (Olympus Deutschland GmbH, Hamburg, Germany) using a 60× water-immersion objective and the appropriate laser lines to capture fluorescent signals. Signals from RNA *in situ* hybridizations (nitro blue tetrazolium chloride/5-Bromo-4-cloro-3-indolyl phosphate precipitate) were imaged with reflection confocal microscopy as described [25]. Images were processed with Imaris 6.4 (BitPlane Inc., Saint Paul, USA) and ImageJ 1.45 software [26]. All image stacks are available [24].

### Bioinformatic tools

Neuropeptide prediction was performed using NeuroPred [27], N-terminal signal peptides were predicted using SignalP 4.0 Server [28]. For multiple sequence alignments, we used ClustalW [29]. The GenBank accession number for the Platynereis RGWamide neuropeptide precursor: JX412226.

## Results

### Generation of specific antibodies against amidated dipeptide epitopes of neuropeptides

**Figure 1:**
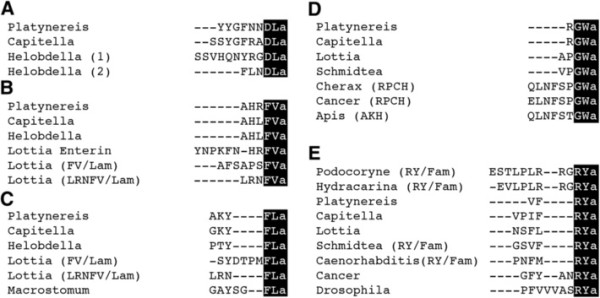
**Two amino-acid amidated motifs are conserved in neuropeptides across phyla.** Multiple sequence alignment of mature neuropeptides with a conserved C-terminus for DLamides (**A**), FVamides (**B**), FLamides (**D**), GWamides (**D**) and RYamides (**E**) from annelids *(Platynereis, Capitella, Helobdella)*, molluscs *(Lottia)*, platyhelminthes *(Schmidtea, Macrostomum)*, nematodes *(Caenorhabditis)*, arthropods *(Cherax, Cancer, Drosophila, Apis)* and cnidarians (*Podocoryne, Hydractinia). *The conserved C-termini that were used for antibody production are highlighted in black. RPCH, red pigment concentrating hormone; AKH, adipokinetic hormone.

We set out to develop antibodies against the conserved C-amidated dipeptides DLa, FVa, FLa, GWa and RYa(‘a’ = ‘amide’) neuropeptides that are conserved across phyla (Figure 1) [23,25-36]. We have recently shown in the marine annelid model *Platynereisdumerilii* that the precursor mRNAs for these neuropeptides are expressed in largely non-overlapping subsets of neurons in the larval episphere. None of these neuropeptides co-expresses with FMRFamide in *Platynereis*[23], suggesting that antibodies against their conserved amidated dipeptides could also substantially increase the number of neurons that can be labelled in other species.

Rabbits were immunized with the short amidated peptides extended with an N-terminal cysteine to allow coupling to a carrier during the immunization procedure. We also used the cysteine residue to couple the peptides to a resin and to affinity purify the antibodies from the respective sera. We employed a high stringency affinity purification protocol including high salt washes and low pH elution to obtain high-affinity antibody fractions.

**Figure 2:**
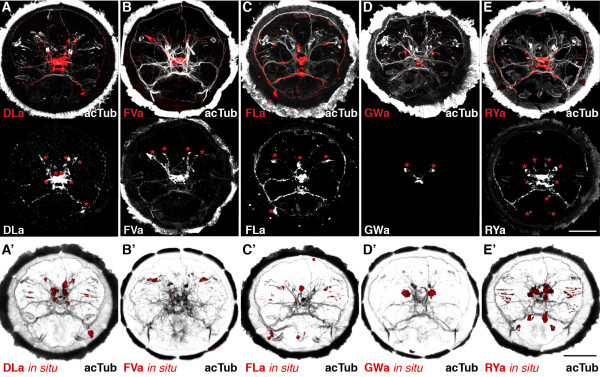
**Correspondence of antibody signals with the respective precursor mRNA expression in *Platynereis. ***(**A-E**) Immunostaining (red) with the DLamide (**A**), FVamide (**B**), FLamide (**C**), GWamide (**D**) and RYamide (**E**) antibodies counterstained for acetylated tubulin (acTub, white) in the upper panels. Bottom panels show immunostainings for the respective antibodies in white. (**A**^**′**^**E **^**′**^) mRNA *in situ *hybridization (red) counterstained for acetylated tubulin (acTub, black) for the DLamide (**A**^**′**^), FVamide (**B**^**′**^), FLamide (**C**^**′**^), GWamide (**D**^**′**^) and RYamide (**E**^**′**^) neuropeptide precursors. With the exception of GWamide, all precursors*in situ* were described in [23] and are shown here for comparison only. All images are anterior views of *Platynereis* larvae 48 h post fertilization (hpf). Asterisks indicate cells that show a spatial correspondence with the mRNA *in situ* hybridization signals in **A**^**′**^**E **^**′**^. Scale bars: 50 μm.

**Figure 3:**
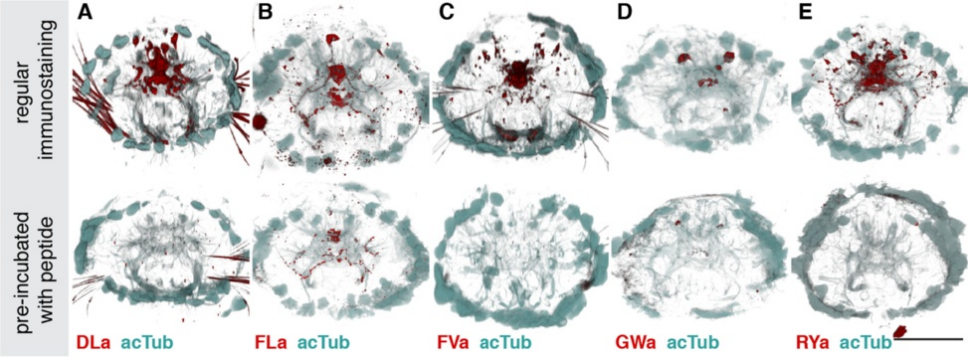
**Blocking of immunostaining signals with peptide pre-incubation. **(**A**-**E**) Regular immunostaining (upper panels), and stainings with antibodies that were pre-incubated with the corresponding synthetic *Platynereis* full length neuropeptide (bottom panels) for DLamide (**A**), FVamide (**B**), FLamide (**C**), GWamide (**D**) and RYamide (**E**), all shown in red. Samples were counterstained for acetylated tubulin (acTub, cyan). All images are anterior views of *Platynereis* larvae 72 h post fertilization (hpf). Scale bar: 50 μm.

Next, we tested the reactivity of the affinity purified neuropeptide antibodies in whole mount immunostainings on *Platynereis *larvae. We found labelling for all antibodies in a subset of neurons and their axons in the larval episphere (Figure 2A-D). To test the specificity of our antibodies, we pre-incubated them in the synthetic amidated full-length *Platynereis* peptides. This treatment led to a complete block of the signal for the anti-DLa, anti-FVa and anti-RYa antibodies (Figure 3A,C,E) and a strong reduction in signal intensity for the anti-FLa and anti-GWa antibodies (Figure 3B,D). These results indicate that the antibodies bind to the respective peptides and this prevents further binding to epitopes in the tissue. The specificity of the antibodies is further supported by the close correlation between the cell body positions revealed by immunostaining and the expression patterns of the respective precursors (Figure 3 A-E, bottom panels, asterisks) as shown by whole-mount RNA *in situ* hybridization (Figure 3 A^′^-E^′^). The recently described antibodies raised against full length *Platynereis *DLa, FVa, FLa and RYa peptides also show very similar neuronal signals [23].

Overall, our specificity tests in *Platynereis* demonstrate that the antibodies raised against amidated dipeptide motifs are remarkably specific and can be used to obtain high-quality tissue stainings. To test the utility of our antibody collection as cross-species-reactive neuronal markers, we performed immunostainings on a variety of marine larvae from different species and phyla.

### DLamide immunoreactivity in annelids

**Figure 4:**
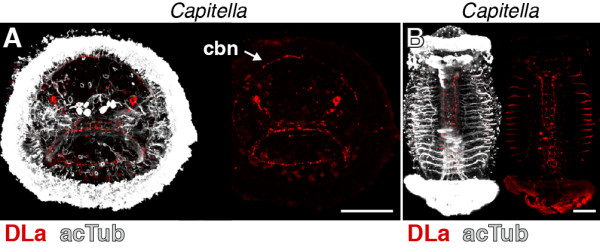
**DLamide immunoreactivity in *Capitella *larvae. **(**A**) Anterior view of an early *Capitella *larva and (**B**) ventral view of a late *Capitella *larva stained with the DLamide antibody (red) counterstained for acetylated tubulin (acTub, white). Scale bars: 50 μm. cbn, ciliary band nerve.

DLa neuropeptides have been described from the errant annelid (Errantia) *Platynereis* and the sedentary annelids (Sedentaria) *Capitella* and *Helobdella*[23,36] (Figure 1A). Since errant and sedentary annelids encompass most of annelid diversity [37], DLa neuropeptides are potentially widely distributed among annelids. To test whether our DLa antibody could be used as a pan-annelid nervous system marker, we also tested its reactivity in *Capitella*. In *Capitella* larvae, we found staining in neurons of the apical organ. These neurons have a flask-shaped morphology typical of sensory cells and project to the larval ciliary band (Figure 4A, arrow) in a similar fashion to that observed for *Platynereis* larvae (compare with Figure 2A). We also observed strong staining in the ventral nerve cord in older *Capitella* larvae (Figure 4B). The specific reactivity of the DLa antibody in both errant and sedentary annelid species demonstrates its usefulness as a pan-annelid neuronal marker.

### FVamide and FLamide immunoreactivity in annelids and molluscs

**Figure 5:**
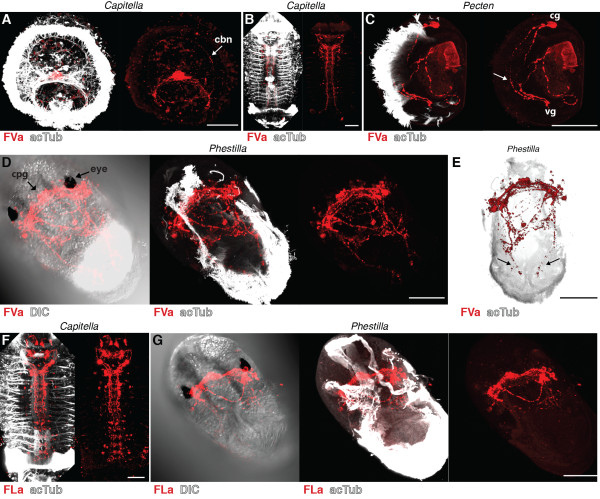
**FVamide and FLamide immunoreactivity in annelid and mollusc larvae.** Immunostainings with the FVamide antibody (red) counterstained for acetylated tubulin (acTub, white) in (**A**) an early *Capitella *larva, anterior view, (**B**) a late *Capitella *larva, ventral view, (**C**) a *Pecten *veliger larva, lateral view, and (**D**, **E**) a *Phestilla *larva, ventral (**D**) and dorsal (**E**) views. Immunostainings with the FLamide antibody (red) counterstained for acetylated tubulin (acTub, white) in (**F**) a late *Capitella* larva, ventral view, and (**G**) a *Phestilla *larva, ventral view. Arrows in (**C**) and (**E**) point at projections that run along the ciliated velum of *Pecten* and the ciliated foot of *Phestilla. *Scale bars: 50 μm. cbn, ciliary band nerve; cg, cerebral ganglion; cpg, cerebropleural ganglion; DIC, differential interference contrast; vg, visceral ganglion.

FVa and FLa neuropeptides have been described in annelids, molluscs and platyhelminths. In the annelids *Platynereis* and *Capitella,* there is one FVa neuropeptide precursor, whereas there are three different precursors in the mollusc *Lottia gigantea* (Figure 1B,C) [35]. FLa peptides are either encoded by a separate precursor gene and expressed in distinct subsets of cells, as in annelids, or co-occur on the same precursor together with FVa peptides, as in molluscs. Regardless of the number of precursor genes, the conserved FVa and FLa epitopes could allow the labelling of all FVa and FLa expressing neurons in annelids and molluscs. We tested the reactivity of both antibodies on *Capitella* larvae and on larvae of the bivalve mollusc *Pecten maximus* and the nudibranch mollusc *Phestilla sibogae* (for morphological details see [38]). In *Capitella,* we found FVa immunoreactivity in apical organ neurons with projections to the ciliary band (Figure 5A, arrow, compare with Figure 2B), and also in the ventral nerve cord (Figure 5B). The FLa antibody labels neurons in the brain and in the ventral nerve cord in older stages of *Capitella* (Figure 5F). In *Pecten* veliger larvae, the FVa antibody labels a small number of neurons in the cerebral and visceral ganglia, some of which project to the ciliated velum (Figure 5C). In *Phestilla,* both antibodies show strong staining in the cerebropleural ganglion between the eyes (Figure 5D,G). The FVa antibody also labels two nerve fibres in the ciliated foot (Figure 5E, arrows).

### GWamide immunoreactivity in annelids, molluscs and crustaceans

**Figure 6:**
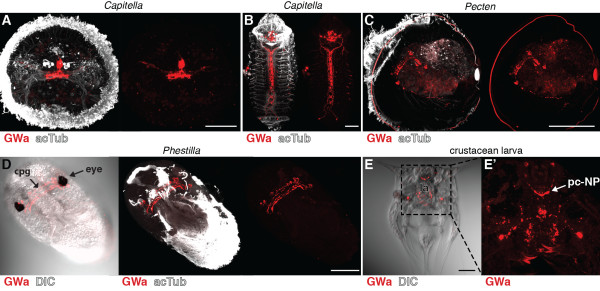
**GWamide immunoreactivity in annelid, mollusc and crustacean larvae.** Immunostainings with the GWamide antibody (red) counterstained for acetylated tubulin (acTub, white) in (**A**) an early *Capitella *larva, anterior view, (**B**) a late *Capitella *larva, ventral view, (**C**) a *Pecten *veliger larva, lateral view, (**D**) a *Phestilla *larva, ventral view, (**E**) and a crustacean larva, ventral view and (**E**^**′**^) a close-up confocal scan. Scale bars: 50 μm. cpg, cerebropleural ganglion; DIC, differential interference contrast; la, labrum; pc-Np, protocerebral neuropil.

GWa neuropeptides are present in annelids, molluscs (APGWa), platyhelminths, crustaceans (as red pigment concentrating hormone, RPCH) and insects (as adipokinetic hormone, AKH, Figure 1D). Although the sequence similarity is limited, the annelid and mollusc GWa precursors are the likely lophotrochozoan orthologues of arthropod RPCH and AKH neuropeptide precursors [39]. We tested our GWa antibody in the annelid *Capitella,* the molluscs *Pecten* and *Phestilla* and a nauplius larva from a cirripede crustacean (for morphological details, see [40]) collected from a plankton sample (Figure 6). Like *Platynereis, Capitella* larvae show staining in a small number of neurons in the apical organ (Figure 6A, compare with Figure 2D) and in the ventral nerve cord of older larvae (Figure 6B), with no ciliary innervation. In *Pecten* veliger larvae, the GWa antibody labels a small number of neurons and their projections (Figure 6C). In *Phestilla,* we found staining in the cerebropleural ganglion between the eyes (Figure 6D). In the crustacean larvae, the antibody labels two cerebral neurons that project to the protocerebral neuropil and a pair of neurons on either side of the labrum (Figure 6E).

### RYamide immunoreactivity in cnidarian, annelid, bryozoan, mollusc and crustacean larvae

RYa neuropeptides have been described in a number of marine phyla, including cnidarians, annelids, molluscs, platyhelminthes and crustaceans. They are also present in terrestrial invertebrates, such as nematodes and insects (Figure 1E). In cnidarians, platyhelminthes and nematodes, RYa peptides co-occur with RFa peptides on the same precursor [33,34,41], whereas in most other phyla they originate from a distinct precursor. Given the broad phyletic distribution of RYa peptides and the observation that they often derive from distinct precursors expressed in different cells than RFa [23], the RYa antibody could have a great value for comparative neuroanatomical studies. To explore the potential of the RYa antibody, we tested its reactivity in cnidarians, annelids, molluscs, bryozoans and crustaceans.

**Figure 7:**
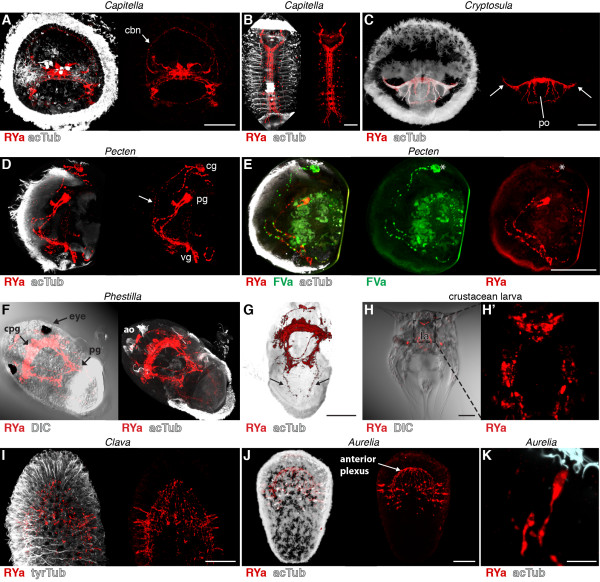
**RYamide immunoreactivity in annelid, bryozoan, mollusc, crustacean and cnidarian larvae. **Immunostainings with the RYamide antibody (red) counterstained for acetylated tubulin (acTub, white) in (**A**) an early *Capitella *larva, anterior view, (**B**) a late *Capitella *larva, ventral view, (**C**) a *Cryptosula* larva, anterior view, (**D**) a *Pecten *veliger larva, anterior view, (**E**) a *Pecten *veliger larva counterstained with the anti-FVamide antibody (green), anterior view, (**F**) a *Phestilla *larva, ventral and (**G**) dorsal view, (**H**) a crustacean larva, ventral view and (**H**^**′**^) a close-up confocal scan. Immunostainings with the RYamide antibody (red) counterstained for acetylated or tyrosylated tubulin (acTub, white) in (**I**) a *Clava *planula larva, lateral view, (**J**) an *Aurelia *planula larva, lateral view, and (**K**) close-up of a sensory neuron of *Aurelia*. Arrows in (**C**) point at axons that project to the ciliary band of *Cryptosula*. Arrows in (**D**) and (**G**) point at projections to the ciliated velum of *Pecten* and the ciliated foot of *Phestilla. *Asterisks in (**E**) indicate neurons that are co-labelled for RYa and FVa. Scale bars: (A-J) 50 μm, (K) 10 μm. ao, apical organ; cbn, ciliary band nerve; cg, cerebral ganglion; cpg, cerebropleural ganglion; DIC, differential interference contrast; la, labrum; pg, pedal ganglion; po, pyriform organ; vg, visceral ganglion.

In *Capitella* larvae, we found RYa staining in individual sensory neurons in the apical organ. As with DLa and FVa, these neurons project to the ciliary band nerve (Figure 7A, arrow, compare with Figure 2E), suggesting a role for RYa neuropeptides in regulating ciliary activity in *Capitella*. We also observed a strong staining in the ventral nerve cord in older *Capitella* larvae (Figure 7B). In larvae of the bryozoan *Cryptosula *species (for morphological details see [42]), we detected strong RYa immunoreactivity in the nerve nodule and in the lateral nerves projecting to the coronal ciliary band (Figure 7C, arrows) and several axons that embrace the pyriform organ. In *Pecten* larvae, we detected two pairs of neurons and their projections along the ciliated velum (Figure 7D, arrow). Using primary antibodies directly pre-labelled with different fluorophores, we also co-stained *Pecten* larvae for RYa and FVa. We observed co-labelling only in a subset of neurons, suggesting that these cells co-express RYa and FVa neuropeptides (Figure 7E, asterisks). This result demonstrates that these antibodies can also be used in combination in a single specimen. In *Phestilla* larvae, we detected a strong RYa signal in the apical organ, the cerebropleural ganglion between the eyes and in the pedal ganglion, as well as in nerves connecting these ganglia (Figure 7F), and also in projections running to the ciliated foot (Figure 7G, arrows). In the nauplius larvae, we found RYa in a group of cerebral neurons that have a flask-shaped morphology and in various neurons surrounding the labrum (Figure 7H). In the cnidarians *Aurelia* and *Clava*, we detected RYa staining in sensory neurons of the ciliated planula larvae, mainly located at the aboral pole (Figure 7I-K). These neurons have sensory morphology with apical sensory dendrites projecting to the surface of the ciliated neuroectoderm (Figure 7K). The basal neuronal projections run along the basal side of the ciliated neuroectoderm and terminate in the anterior plexus. These results show that the RYa antibody is a neuronal marker widely applicable across several invertebrate phyla. It should be noted that the RYa antibody may cross-react with invertebrate neuropeptides belonging to the NPF/NPY (short neuropeptide F, NPF; short neuropeptide Y, NPY) family, that sometimes have a C-terminal RYa, such as in *Apis mellifera* and *Bombyx mori* NPFs [43].

## Discussion

### Amidated dipeptide epitopes allow the generation of specific antibodies

**Figure 8:**
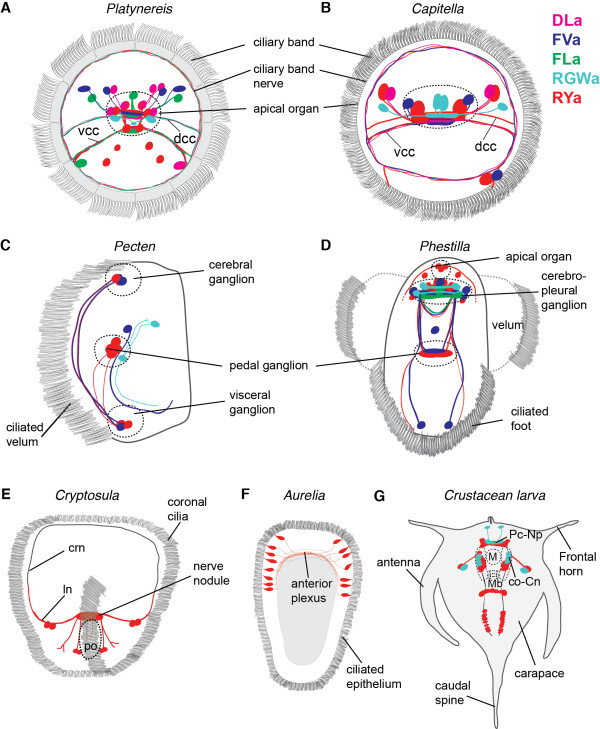
**Summary diagrams of peptidergic cells in various species. **Summary diagrams showing the position of peptidergic cells labelled with our cross-species reactive neuropeptide antibodies for *Platynereis*, anterior view (**A**), *Capitella*, anterior view (**B**), *Pecten*, lateral view (**C**), *Phestilla*, ventral view (**D**), *Cryptosula*, anterior view (**E**), *Aurelia*, lateral view (**F**) and the crustacean larva, ventral view (**G**). co-Cn, circumoral connective; crn, coronal ring nerve; dcc, dorsal branch of the circum-oesophageal connectives; ln, lateral nerve; m, mouth; mb, mandible; Pc-Np - protocerebral neuropil; po, pyriform organ; vcc, ventral branch of the circum-oesophageal connectives.

We have shown, using a variety of species, that our antibodies against amidated dipeptides can be used to label distinct subsets of peptidergic neurons (Figure 8, confocal stacks are available [24]). Our finding that several different amidated dipeptides could be used to generate specific antibodies broadens our understanding of the immunogenic potential of peptide sequences. It is interesting to note that the company we contacted for antibody production initially warned us not to carry out the project, arguing that ‘It is considered that up to 5aa peptides are not immunogenic at all.’ The strong immunogenicity of these peptides must be due to their C-terminal amidation, in the context of the two residues. It is not the amide group that is recognized alone, since all peptides have it, yet we see no cross-reactivity. The importance of amidation, and not the two amino acids alone, is supported by the observation that such dipeptide motifs can be found in thousands of other proteins (for example, 61,717 DL, 32,582 FV, 9,459 GW and 18,792 RY in the *Capitella* predicted proteome), yet we do not see strong background staining in our immunostainings on many different invertebrate species.

Overall, our data argue that the antibodies strongly and specifically bind the amidated peptides we used for immunization. First, the stringent affinity purification protocol we employed together with the peptide-blocking experiments indicates that the antibodies strongly bind to the short amidated peptides. Second, the specific neuronal stainings in tissues corresponding to the expression patterns of the precursor genes in *Platynereis* show that the antibodies specifically bind to the respective peptides.

The strategy we employed to generate specific cross-species antibodies could also be applied to other conserved neuropeptides present in diverse taxa. With the increasing sampling of metazoan genomes and transcriptomes, and the accumulation of data from understudied groups (for example, hemichordates, platyhelminths, priapulids), we will have the chance to identify further conserved peptide motifs. Further sampling will also allow the identification of other taxonomic groups in which the antibodies described here could be used as neuronal markers. Given the brevity of the sequences, reactivity to multiple neuropeptide families with the same amidated termini cannot be excluded. For the proper interpretation of staining patterns, it is therefore also important to study mRNA expression and to scrutinize available transcriptomic and genomic resources. Importantly, our results show that these antibodies are not widely cross-reactive and do not recognize other amidated peptides. A single amino acid change seems to be sufficient to prevent antibody binding, since the DLa, FLa and FVa antibodies all recognize different cells.

Finally, C-terminal amidation is commonly used for immunization for peptides that derive from an internal part of the protein, to keep the peptide closer to its natural state. Our results caution that such an unnatural terminal amide in internal peptide sequences may trigger an undesired immune response, and potentially cause cross-reactivity to naturally occurring amidated peptides.

### Cross-species antibodies suggest that the neuropeptidergic control of cilia is widespread in marine larvae

With the DLa, FVa and RYa antibodies, we commonly observe projections to larval ciliary bands. We have recently shown that the neurons expressing these neuropeptides also innervate the ciliary band in *Platynereis* larvae, and that these peptides regulate the activity of cilia. All three peptides increase the beating frequency of cilia and inhibit ciliary arrests, thereby influencing the swimming depth of planktonic *Platynereis* larvae [23]. In *Capitella* larvae, all three neuropeptides are present in the ciliary band nerve. In *Pecten,* we found FVa and RYa immunoreactivity in nerves running along the ciliated velum, and in *Phestilla* in projections in the ciliated foot*.* RYa neurons also seem to innervate locomotor cilia in bryozoan and cnidarian larvae. This suggests that these peptides may also regulate ciliary activity in these larvae, indicating a general role for neuropeptides in the regulation of ciliary locomotion in marine invertebrate larvae [44].

## Conclusions

We developed specific cross-species reactive antibodies that recognize the conserved neuropeptide motifs DLamide, FVamide, FLamide, GWamide and RYamide. These antibodies can be used in a wide range of marine invertebrates, including annelids, molluscs, bryozoans and cnidarians. Further genomic and transcriptomic sampling could identify other animal groups where these peptide motifs are conserved and where our antibodies could also be employed. Our work also highlights the antigenic potential of very short amidated peptide motifs. The ongoing sampling of neuropeptide diversity will allow the development of other similar antibodies, to enrich further the comparative neurobiology toolbox. Our sampling across diverse marine larvae demonstrates the broad utility of these antibodies, and also indicates that the neuropeptidergic regulation of ciliary locomotion may be a general feature of marine ciliated larvae.

## Abbreviations

acTub: Acetylated tubulin; AKH: Adipokinetic hormone; ao: Apical organ; cbn: Ciliaryband nerve; cg: Cerebral ganglion; co-Cn: Circumoral connective; cpg: Cerebropleural ganglion; crn: Coronal ring nerve; Dcc: Dorsal branch of the circum-oesophageal connectives; DIC: Differential interference contrast; Hpf: Hours post fertilization; la: Labrum; ln: Lateral nerve; m: Mouth; mb: Mandible NPF; F: Short neuropeptide; NPY: Short neuropeptide; Pc-Np: Protocerebral neuropil; pg: Pedal ganglion; po: Pyriform organ; RPCH: Red pigment concentrating hormone; tyrTub: Tyrosylated tubulin; vcc: Ventral branch of the circum-oesophageal connectives; vg: Visceral ganglion.

## Competing interests

A patent application has been submitted.

## Authors’ contribution

MC carried out antibody purification, tissue stainings and imaging and wrote the paper. GJ conceived the study, participated in its design and wrote the paper. Both authors read and approved the final manuscript.
